# Primary care physicians' attitudes, practices, and perceived barriers toward depression screening in older people in the Kingdom of Bahrain

**DOI:** 10.3389/fmed.2024.1403469

**Published:** 2024-11-06

**Authors:** Fatema Habbash, Zainab Khamis, Zahra Alasfoor, Aayat Mahdi, Masooma Alsharkhat, Noor Hasan, Alaa Al Qari, Sadok Chlif, Amer Almarabheh, Afif Ben Salah

**Affiliations:** ^1^Department of Family and Community Medicine, Arabian Gulf University, Manama, Bahrain; ^2^Department of Family Medicine, University Medical Center King Abdullah Medical City Bahrain, Manama, Bahrain; ^3^Clinical Master in Family Medicine Program, Arabian Gulf University, Manama, Bahrain; ^4^Primary Health Care Centers, Manama, Bahrain; ^5^Institute Pasteur de Tunis, Tunis, Tunisia

**Keywords:** older people, primary care physician, depression, mental health, screening, attitudes, practice, barriers

## Abstract

**Purpose:**

This study aimed to explore attitudes, practices, and perceived barriers of primary care physicians (PCPs) toward depression screening in older people.

**Methods:**

This cross-sectional study enrolled PCPs from randomly selected representative primary care centers in Bahrain. A self-administered piloted semi-structured questionnaire was used for data collection.

**Results:**

We enrolled 248 PCPs in the study (82.3% females, and the mean age = 40 ± 8.7 years). More than half of the participants (54.4%) had a positive attitude toward depression screening in older people. However, only 10.9% of the participants reported systematically screening for this condition without using specific tools for screening in 45.5%. The most reported barriers toward this service are short consultation time (95%), the presence of multiple co-morbidities in this age group (90%), and the absence of guidelines or appropriate training in around 30%. Positive attitudes were significantly higher among older PCPs (*p* = 0.039), family physician consultants (*p* = 0.008), those with more than 10 years of work experience (*p* = 0.024), and those who participated in related educational activities (*p* = 0.007). Under-screening practice is associated with perceived short consultation time (p = 0.002), insufficient continuous medical education (CME) activities attendance in older people's mental health (p = 0.048) as well as having a general physician's title (*p* = 0.049). Only the PCPs' job title, Adjusted Odds Ratio (OR) ^=^ 3.513, 95 C.I [1.225-10.074] and attendance of CME activities, OR = 1.278, 95 C.I [1.098 – 3.192] remained significant when controlled for age and experience.

**Conclusion:**

More training on older people's mental health and provision of screening and management guidelines are priorities to promote older people's mental health in primary care settings.

## 1 Introduction

Aging of the global population creates significant medical and sociodemographic problems worldwide (1). According to the World Health Organization (WHO), by the year 2050, the percentage of older people over 60 will increase from 12% to 22% (2). In Bahrain, the population aged 65 and above accounted for 2.8% in 2018 (3). As the population ages, it is crucial to ensure and maintain equitable access to health care including disease prevention, treatment, and rehabilitation (3). The WHO identified a gap in mental health prevention and management and developed pertinent content to support post-graduate primary health care training (4, 5). Depression is the most common mental health problem in older people, leading to considerable morbidity and mortality rates, in contrast to younger patients, older people with depression are more likely to have several comorbidities and cognitive impairment, which might contribute to suboptimal diagnoses and treatment (6).

Globally, the estimated pooled prevalence of depression among older people was 28.4% (7). Studies in the MENA regions revealed depression rates among older people ranging from 13% to 96.7% (7–12). In Bahrain, a recent study indicated an alarming proportion of 50.6% of depression among older people over the age of 60 (13). The estimated prevalence of depression among Bahraini older adults attending primary healthcare centers in 2009 was 41.5% (14).

Primary care physicians (PCPs) are usually the first medical contact with older patients and have a significant role in the early detection, and management of depression, and prevention of impairment and suicide (15). While the US Preventive Task Force (USPTF) recommends that all individuals older than 60 years should be screened for depression periodically (16, 17), the literature revealed a variation in PCPs' attitudes and practices toward depression screening and management in older patients (18–20). The reluctance of PCPs to discuss depression with older patients may be attributed to the desire to avoid feelings of powerlessness when therapeutic options appear limited (21, 22). Stigmatizing attitudes are also commonly reported by PCPs toward people with mental disorders which can hinder addressing the psychological components of the condition and initiating therapy (21, 23). While most PCPs in different contexts might have positive attitudes toward depression screening in older adults, it was reported that it was sometimes not reflected in their practice (2, 24). More than one decade ago, a study conducted in Bahrain, reported that only 10.6% of PCPs have been always screening for depression in older patients (25). The findings in the literature indicate that PCPs who were older, more experienced, attended mental health educational activities, and had frequent encounters with cases of older people in their practice have significantly better attitudes toward the screening and management of depression in older patients (2, 24, 25).

Depression is a major health problem with a heavy burden and impact on quality of life (13, 16, 26). Previous studies revealed that despite the high prevalence of depression among older patients visiting primary care centers in Bahrain, the screening for such problems by PCPs is suboptimal (14, 25). We conducted this study as part of a quality improvement research project to explore PCPs' attitudes, practices, and perceived barriers toward depression screening in older people. The findings of this study will contribute to optimizing the mental health care services provided for this age group by implementing evidence-based interventions at the primary care level.

## 2 Materials and methods

### 2.1 Study design and variables

We conducted a cross-sectional analytical study to determine factors associated with PCPs' attitudes and practices toward older people's depression screening as outcome variables. We also analyzed perceived barriers by PCPs toward depression screening in older people to identify potential areas for improvement.

Ten statements were used to assess PCPs' attitudes, five statements represent positive attitudes and the other five represent negative attitudes using a Likert scale from one to five. Participants who scored above the mean score of attitude statements were considered to have a “positive attitude”. The independent variables include PCPs' sociodemographic, qualifications, training, and job-related factors.

### 2.2 Study population and setting

The target population was PCPs working in governmental primary care centers in Bahrain. PCPs working in primary care centers in Bahrain can be either family physician consultants who are practicing doctors in primary care centers with postgraduate training and specialization in primary care with a minimum of 4 years of experience, family physician specialist who holds qualifications similar to the consultant, but with < 4 years of experience, or general physician who are practicing doctors in primary care centers with no postgraduate training and specialization in primary care.

The study sample is calculated using the formula for the simple random sampling approach, where Z = 1.96, *P* = 0.5, E= margin of error = 0.05, and *N* (population size) = 404. The total estimated sample size was around 200. We increased the sample size by 25% to compensate for potential non-respondents.

### 2.3 Sampling method and study tool

There are five health regions in Bahrain with an unequal number of PCPs in each region. The number of targeted PCPs in each health region was proportional to PCPs working in that region. Three to five health centers were randomly selected from each of the five health regions to ensure that the required number of PCPs from each region is included in the sample. All PCPs in the selected health centers were eligible to participate in the study, except for family physicians under training programs or those who did not provide written informed consent. The human resources director provided the list of all eligible PCPs to whom the study team distributed a piloted self-administered questionnaire. An information sheet explaining the study was provided to all eligible participants. All participants provided written informed consent before enrolment.

The questionnaire is composed of several sections to collect data on PCPs' sociodemographics, job experience, qualifications, and practice toward older people's depression screening (symptoms that trigger screening, screening tools, and frequency of screening) a list of multiple choices was provided and an open text to elaborate if needed. The participants' attitude toward older people's depression screening was evaluated by providing a list of statements, based on the work of Glasser et al. (2) and Ghzwany et al. (22), in which the participant can respond by their degree of agreement on a Likert scale (strongly disagree, disagree, neutral, agree and agree strongly). Examples of the statements include “I find it difficult to discuss emotional issues with older people”, “I avoid using the term depression to avoid stigma” and “It is my responsibility to screen and diagnose an older individual for depression”. The perceived barriers were evaluated by asking the participants to respond to a list of possible barriers as we found in the literature answers are not mutually exclusive as well as open text to elaborate on any other barriers and their perspectives on recommendations to improve older people's depression screening.

### 2.4 Statistical analyses

We entered data, from validated filled questionnaires, into Excel referring to a codebook, and identifying the variable type, name, and code. We cleaned and checked the electronic data documented before the export to Statistical Package for the Social Sciences (SPSS) software version 28.0.1.1. Categorical variables are presented as frequencies and percentages, and continuous variables as means and standard deviations. The χ^2^ test tested the association between categorical variables considered as predictors of PCPs' attitudes and practice toward depression screening in older people as outcome variables. A logistic regression model permitted to estimation of the adjusted odds ratios and their 95% confidence limits of predictors of positive attitudes. A *p*-value of < 0.05 was considered statistically significant.

### 2.5 Ethical considerations of the study

The study protocol was approved by the Research and Ethics Committee at the College of Medicine and Medical Science at Arabian Gulf University (approval number: E09-PI-11-22) and by the Research and Ethics Committee in Primary Health Care in the Kingdom of Bahrain. Participation in the study was voluntary and consent was obtained from participants before filling out the survey. The invited participants were informed that their identity would not be disclosed and their refusal to participate would be confidential to avoid any source of coercion. The findings from the study will be used exclusively for the quality improvement of this service.

## 3 Results

### 3.1 Characteristics of the study sample

A total of 248 PCPs out of 252 invited from eligible health centers participated in the study (response rate= 98.4%). The mean age of the participants was 40 ± 8.7 years, the majority were females (82.3%), and most were family medicine consultants (45.65%) and specialists (44.4%). This distribution reflects the national statistics of primary care physicians in The Kingdom of Bahrain. The mean duration of work for the physicians was 10.5 ± 8.9 years. Three-quarters (75%) of the physicians reported that 25% or more of their patients were over the age of 60. Nearly half (53.6%) of the physicians reported attending continuous medical education (CME) activities related to older adult mental health. We noticed that only 27 PCPs (10.9%) reported that they always screen for depression among older people, while 209 (84.3%) screen occasionally and 12 (4.8%) never screen for this condition. Most PCPs (241, 97%) reported that loss of interest and pleasure by older patients is the most common symptom that triggers physicians to screen for depression, while the least symptoms were sexual complaints and pain which were reported by 161 (63%) and156 (65%), respectively. Surprisingly, 112 out of 248 PCPs (45.5%) reported not using any depression screening tool. The PCPs might use more than one tool for screening patients. For those who reported using such screening tools (132 PCPs), the most frequently used is PHQ-2 (50.7%) compared to the Mini-Mental State Exam (40.3%) or the Geriatric Depression Scale (32.1%). Table 1 summarizes the sociodemographic characteristics and the practice of the study participants toward depression screening in older people.

**Table 1:** Characteristics of the study participants and depression screening practices in older people practices (*n* = 248).

**Variable**	***N* (%)**
**Physician gender**	
Male	44 (17.7)
Female	204 (82.3)
**Age**	39.73 ± 8.73
< 35 years	91 (37.2)
35– < 45 years	88 (35.9)
≥45 years	66 (26.9)
**Current job title**	
Family physician consultant	113 (45.6)
Family physician specialist	110 (44.3)
General physician^a^	25 (10.1)
**Experience**	
< 5 years	85 (34.3)
5–10 years	65 (26.2)
>10 years	98 (39.5)
**% of patients** >**60 years in clinic**^b^	
< 25%	62 (25)
26–49%	162 (65.3)
≥ 50%	24 (9.7)
**Attended CME or conferences**	
No	115 (46.4)
Yes	133 (53.6)
**Frequency of screening of depression in older patients**	
Never	12 (4.8)
Sometimes	209 (84.3)
Always	27 (10.9)

^a^General physician: a practicing doctor in primary care centers with no postgraduate training and specialization in primary care.

^b^Estimated percentage of patients over 60 years in their practice.

### 3.2 Primary care physicians' attitude and perception regarding the diagnosis and management of depression in older adults

One hundred thirty-five (54.4%) of the participated PCPs have a mean score above the cutoff of median scores (> 36 out of 50) thus considered as having positive attitudes toward screening and management of depression in older adults.

The PCPs rated the different items of attitudes as follows: 86% agreed that it is their responsibility to recognize depression in older adults; more than half (57%) were confident in diagnosing depression while 61% reported that they do not focus on diagnosing depression until excluding organic causes. The majority of PCPs (91%) believe that family members need to be engaged in the older patient's care and management in cases of depression. In terms of management, only 36% of physicians feel confident about prescribing antidepressants to older patients, while 43% prefer to refer their patients to a psychiatrist.

### 3.3 Primary care physicians reported barriers regarding screening and management of depression in older patients

**Figure 1:**
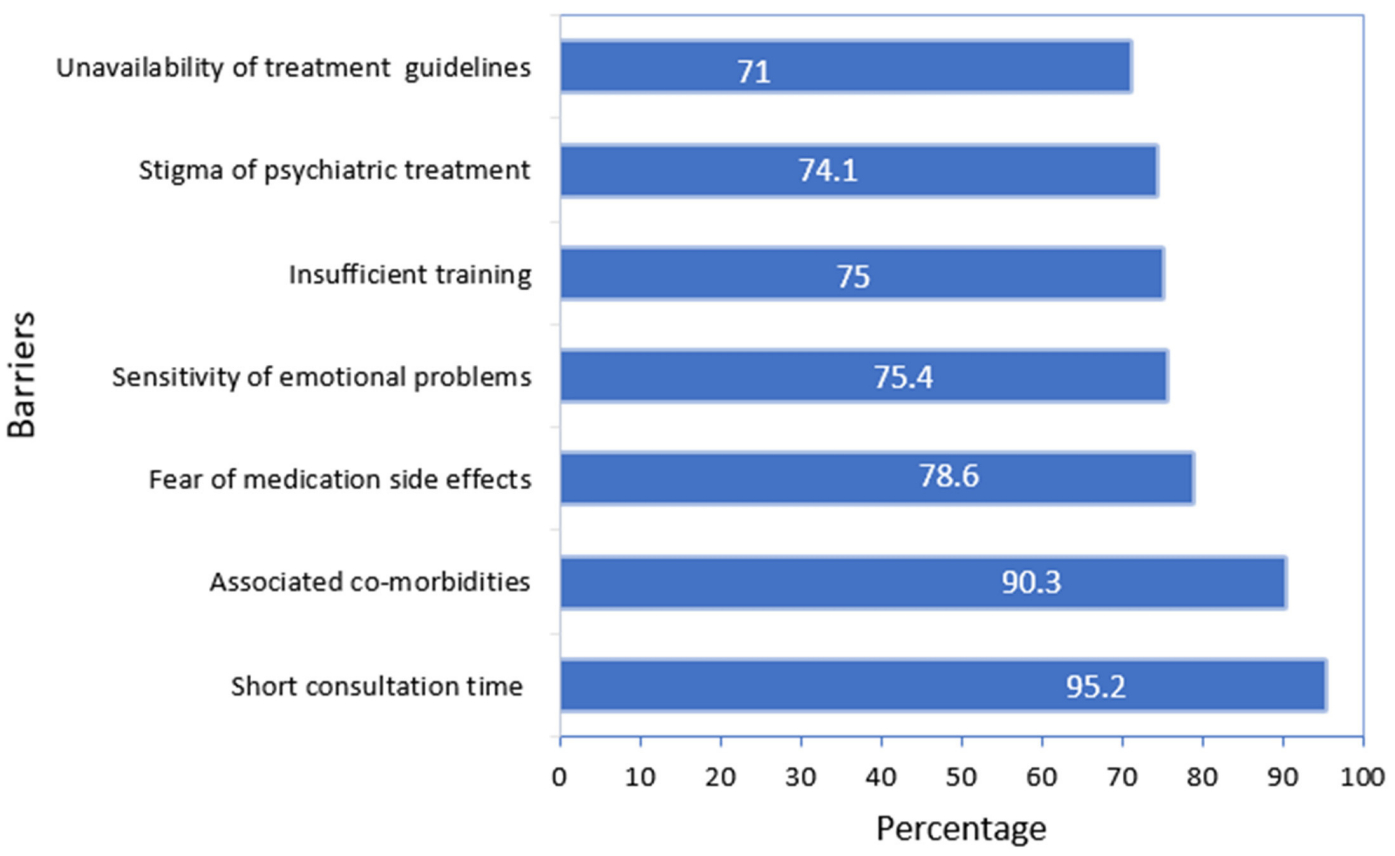
Primary care physicians' reported barriers toward geriatric depression screening.

The most reported barriers toward screening and management of depression in older patients were short consultation time (95%) and patients with multiple co-morbidities (90%). Around two-thirds of PCPs reported other barriers related to insufficient confidence because of the absence of guidelines and appropriate training, all of those represented in Figure 1.

### 3.4 Factors associated with physicians' attitudes toward depression screening in older people

Bivariate analyses revealed that positive attitudes toward depression screening in older people and management were significantly higher among PCPs who were older than 35 years (*p* = 0.039), family physician consultants (*p* = 0.008), those who have more than 10 years of work experience (*p* = 0.024), and those who reported participating in older people depression screening and management CME activities (*p* = 0.007). There was no significant association with gender or the reported percentage of older patients in their practice (Table 2).

**Table 2:** Association of primary care physicians' attitude toward depression screening in older people with participants characteristics and practice.

**Variable**	**Attitude**		***P* value**
	**Negative** ***n*** **(%)**	**Positive** ***n*** **(%)**	
**Gender**			
Male	18 (40.9)	26 (59.1)	0.617
Female	93 (46)	109 (54)	
**Age**			
< 35 years	50 (54.9)	41 (45.1)	0.039
35– < 45 years	39 (44.3)	49 (55.7)	
45 years and above	22 (34.4)	42 (65.6)	
**Job title**			
Family physician consultant	39 (35.1)	72 (64.9)	0.008
Family physician specialist	56 (50.9)	54 (49.1)	
General physician	16 (64)	9 (36)	
**Experience**			
< 5 years	48 (56.5)	37 (43.5)	0.024
5–10 years	28 (43.1)	37 (56.9)	
>10 years	35 (36.5)	61 (63.5)	
**Attended CME**			
No	62 (54.4)	52 (45.6)	0.007
Yes	49 (37.1)	83 (62.9)	
**Average percentage of patients**			
< 25%	26 (41.9)	36 (58.1)	0.269
26–49%	71 (44.1)	90 (55.9)	
More than or equal to 50%	14 (60.9)	9 (39.1)	
**Frequency of screening for geriatric depression**			
			0.034
Sometimes	104 (47.5)	115 (52.5)	
Always	7 (25.9)	20 (74.1)	

The multivariate analysis by logistic regression model confirmed that only the PCPs' job title, Adjusted Odds Ratio (OR) = 3.51, 95 C.I [1.22–10.07] and attendance of CME activities, OR = 1.27, 95 C. I [1.09–3.19] remained significant when controlled for age and experience (Table 3).

**Table 3:** Predictors of primary care physicians' positive attitudes toward depression screening in older people.

**Characteristics**	**OR^a^**	**95% C.I.^b^**	***P* value**
**Age group**			
< 35 years	Ref.		
35–45 years	0.94	0.38–2.28	0.894
≥45 years	1.53	0.47–4.98	0.479
**Job title**			
General physician	Ref.		
Family physician specialist	2.617	0.93–7.34	0.068
Family physician consultant	3.513	1.22–10.07	0.019
**Attended CME or conferences**			
No	Ref.		
Yes	1.872	1.09–3.19	0.021
**Experience**			
< 5 years	Ref.		0.571
5–10 years	1.289	0.53–3.09	0.696
> 10 years	1.278	0.37–4.38	

OR ^a^Adjusted odds ratio, ^b^Confidence interval.

Regarding screening for depression among older patients, we noticed that only 10.9% of PCPs reported that they always provide this service. Under screening practice is associated with perceived short consultation time (*p* = 0.002), reporting insufficient CME attendance in older people's mental health (*p* = 0.048) as well as having a family medicine consultant or specialist compared to general physician's job title (*p* = 0.049).

### 3.5 Primary care physicians' recommendations regarding screening and management of depression in older adults

**Figure 2:**
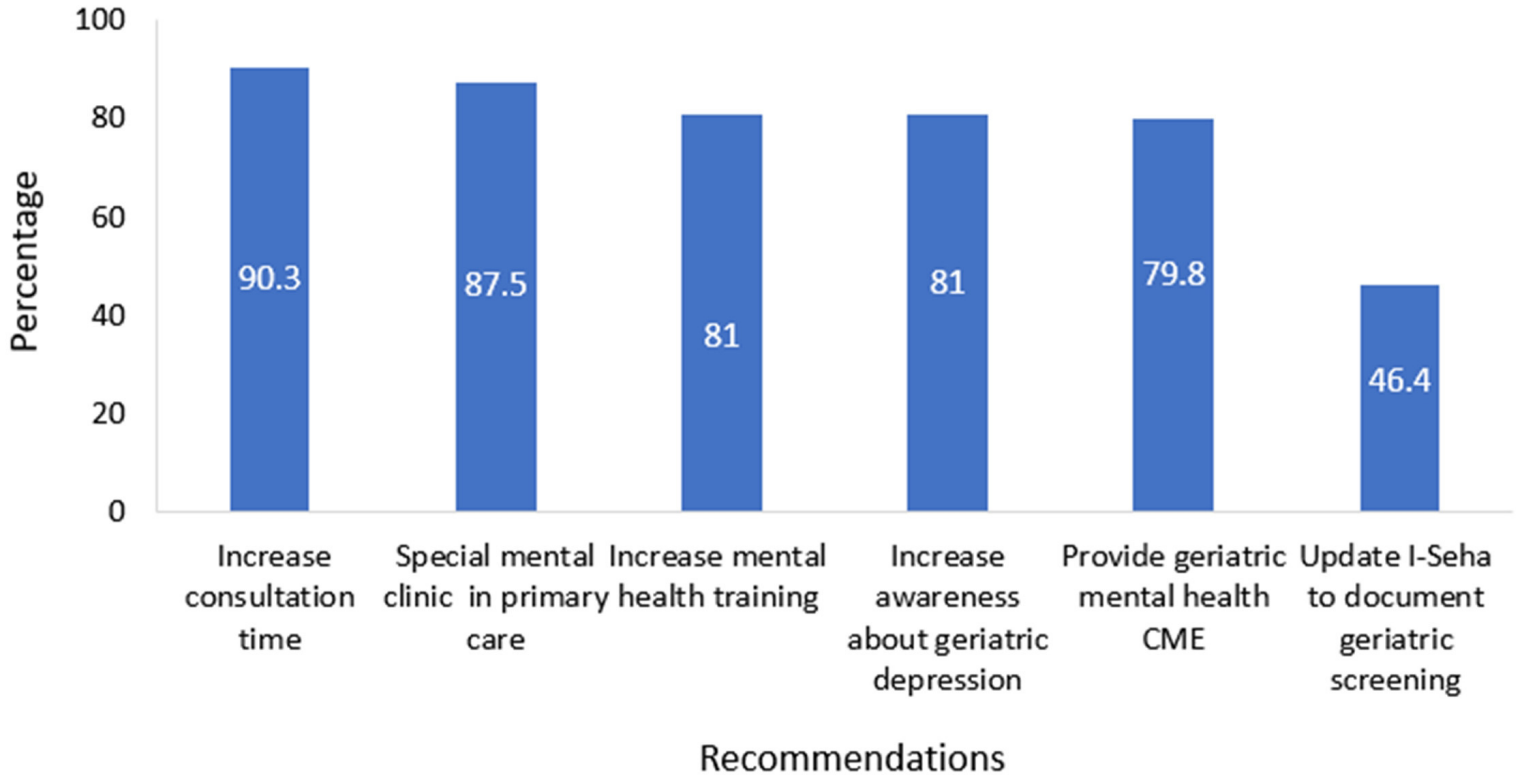
Main recommendations for improving depression screening in older people.

We explored, from PCPs' perspective, the future needs and directions for improving depression screening and management in older people. Primary care physicians' recommendations regarding screening and management of depression in older adults are presented in Figure 2. Two hundred and twenty-four physicians (90.3%) reported that increasing consultation time for older patients and 217 (87.5%) recommended the establishment of special mental health clinics that will provide care to older individuals in primary care settings. Moreover, 201 (81%) recommended that mental health training should be emphasized in residency and training programs and 201 (81%) also suggested increasing awareness campaigns about depression in older people in the community. One hundred and ninety-eight (79.8%) recommended arranging CME activities on older people's mental health. One hundred and fifteen (46.4%) proposed structuring electronic medical records to make depression screening in older people mandatory in primary health care consultation.

Other recommendations included the implementation of a mental health consultation for older people in primary health care services and setting specific indicators of performance. The study participants also emphasized the importance of daycare centers and activities to engage older people, establish support groups for depressed older individuals, and provide scholarships for PCPs to specialize in geriatric medicine and mental health.

## 4 Discussion

We conducted a cross-sectional study among a representative sample of PCPs in Bahrain to decipher their perspectives and practices toward depression screening in older people. More than half of the PCPs who participated in this study had a positive attitude toward depression screening in older people. However, only 10.9% of the participants reported systematically screening for depression in older patients, and nearly half of PCPs not using specific screening tools. The main barriers reported are organizational (limited time for consultation, absence of appropriate treatments in the setting or lack of documentation in the electronic medical record system “Iseha”), related to training (Lack of guidelines and CME related contents, fear of drugs' side effects, patients with multiple morbidities) or psychological factors (sensitive questions and stigma). Experience and specific training in mental health are the predictors of screening for this condition. PCPs expressed the need for more training and provision of management guidelines and medications for depression in primary health care centers.

Studies conducted in different contexts are consistent with this study's findings related to the positive attitudes of PCPs toward older people's depression screening (2, 19, 24, 27, 28). For instance, PCPs in other contexts recognized that screening and diagnosing depression in older people is one of their responsibilities (2, 19, 24, 28). However, only 26% of PCPs in this study reported that they avoid using the term “depression” to avoid the risk of stigma, which is lower than what was reported in Saudi Arabia, where 48% of the study participants of PCPs reported avoiding using the term/diagnosis “depression “as it was linked with stigma (2). The stigma associated with having a mental illness might negatively influence the seeking of healthcare services among older adults with depression, therefore, it could hinder early diagnosis and interventions by healthcare providers (29). There are cultural variations of stigmatizing attitudes and perceptions of mental health problems (30). Innovative strategies to enhance community awareness regarding mental health problems, particularly among older adults with depression, can mitigate the internalized stigma perceived by patients and external stigma by the community and healthcare providers to enhance the early detection and management of this condition (31).

The present study showed that PCPs' positive attitudes toward depression screening in older people were associated with older age of the PCP, higher qualification, more years of experience, and participation in CME activities related to older people's mental health, which is consistence with other studies (24, 28). In fact, the multivariate analysis confirmed that only the PCPs' qualification and participation in older people's mental health CME activities are independently predicting the PCPs' positive attitudes toward depression screening in older people after controlling for age and years of experience. These findings confirm that training in older adults' health including mental health aspects at different phases and through innovative approaches, could have a significant impact on PCPs' attitudes and practices toward depression screening in older people and enhance patient satisfaction and trust in the older people quality of care at the primary care setting (18, 32).

Interestingly, although most PCPs who participated in this study had positive attitudes toward depression screening in older people, it was not consistent with their practice, as we found that only 10.9% of the participants reported always screening for depression in older adults, and nearly half of the participants do not use any specific tool for screening. Surprisingly, our finding reiterated the results of a study conducted in the primary care setting in Bahrain more than a decade ago and found that only 10.6% of PCPs were routinely screening for depression in older adults (25). This result confirms the persistence of this gap and the urgency to implement a quality improvement project to address it. Other findings in the literature are inconsistent regarding PCP's practices toward depression screening in older people, while some indicate optimal screening and use of proper screening tools, others reported a gap in this aspect (2, 18, 19, 24, 27, 28). This could be explained by the impact of culture regarding mental health problems, in addition to variations in PCPs' training and system-related factors such as consultation time and exposure to continuous professional developmental activities.

The novel aspect of this study is exploring the barriers, from PCPs' perspective, that hinder routine depression screening in older people and recommendations to overcome these barriers within the primary care setting. The most reported barriers and recommendations to optimize depression screening in older people are organizational or related to insufficient specific training. Indeed, PCPs are facing many complexities when dealing with older patient's mental healthcare. These are related to psychiatric, medical, and neurologic disorders, functional loss that are common in this age group as well as social, cultural, and economic dimensions (33). Due to the shortage of physicians trained in late-life mental health, most older individuals will continue to receive sub-optimal health care. Education and training in the essentials of older people's mental healthcare are required and highly needed during undergraduate medical school, as well during residency and specialization programs and ongoing professional development activities for PCPs. Avoidance of stereotyping, effective communication, and strategies for ensuring comprehensive healthcare that includes mental health for older adults are areas of training priorities for PCPs (34). This training can enhance the knowledge, skills, and perspectives required to address current and future challenges in caring for older adults with mental disorders (33). Furthermore, PCPs can benefit from inter-professional education that overcomes professional silos and enables them to collaborate in interdisciplinary, team-based models of mental health care (34).

Although PCPCs in our study recommended the establishment of special mental health clinics that provide care to older individuals in primary care settings, such intervention might not be sustainable and could contribute to the stigmatization and disintegration of primary health care services. Innovative approaches in primary health services such as collaborative-interprofessional mental health care that deliver person-centered, integrated care responsive to all age groups including older people, by trained and skilled healthcare workers are required to enhance optimal management of this health problem (34).

To the best of our knowledge, this study is the first in Bahrain to investigate factors affecting PCPs attitudes and practices and explore barriers and recommendations from their perspectives toward depression screening in older adults. Despite the large and representative sample size (≈ 61% of the PCPs and 70% of health centers), it suffers from some limitations. First, using a self-administered questionnaire for data collection could lead to response and recall biases. In addition, a social desirability bias could occur because the study was conducted by family physician specialists under training. All these limitations justify future studies, to evaluate mental health and to investigate mental health problems burden and their impact on quality of life from the lens of older people. Triangulating information from different stakeholders including healthcare providers and older people, using mixed methods studies, would provide a comprehensive evaluation while also engaging and enabling older people to accelerate the implementation of more age-friendly healthcare settings that consider older people's holistic wellbeing (3).

## 5 Conclusion

The findings of this study are instrumental in indicating high-priority areas for interventions to improve PCP screening, diagnosis, and management of depression in older people in primary care settings in Bahrain. These interventions should include structured and innovative approaches to training on older people's mental health and the provision of screening and management guidelines to promote older people's mental health in primary care settings. In addition, the findings of this study support the establishment of sustainable national programs addressing older people's mental health needs, a critical step toward meeting the UN Decade of Healthy Aging's goal of improving the lives of older people and families (3).

## Data Availability

The raw data supporting the conclusions of this article will be made available by the authors, without undue reservation.
